# More Pain, More Gain! The Delivery of COVID-19 Vaccines and the Pharmaceutical Industry’s Role in Widening the Access Gap

**DOI:** 10.34172/ijhpm.2022.6942

**Published:** 2022-08-20

**Authors:** Luciana Correia Borges, Henrique Zeferino de Menezes, Eric Crosbie

**Affiliations:** ^1^School of Public Health, University of Nevada Reno, Reno, NV, USA.; ^2^Department of International Relations, Federal University of Paraiba, João Pessoa, Brazil.; ^3^Ozmen Institute for Global Studies, University of Nevada Reno, Reno, NV, USA.

**Keywords:** Pharmaceutical Industry, COVID-19 Pandemic, COVID-19 Vaccine, COVAX Facility, Human Rights, World Health Organization

## Abstract

**Background:** An effective response to the coronavirus disease 2019 (COVID-19) pandemic entails a comprehensive strategy that ensures equitable access to all COVID-19-fighting technologies. To achieve this goal, the international community has acknowledged immunization as a public good. However, a trend of grossly unequal dose distribution emerged, owing, among other factors, to pharmaceutical companies’ profit-driven actions, jeopardizing the mechanisms built to increase vaccine access. The contradiction between public health interests and corporate discretion in determining vaccine dose distribution poses critical concerns about the health risks associated with lengthening the duration of the pandemic and the eventual liability of companies for violations of human rights.

**Methods:** To evaluate the risks posed to the COVID-19 immunization program, data on vaccine allocation and delivery, vaccine dose application, immunized populations, and the volume of Advanced Purchase Agreements (APAs) between countries and pharmaceutical companies were compiled and assessed. A descriptive analysis was then conducted to analyze the role of pharmaceutical companies in providing equitable access to COVID-19 vaccines.

**Results:** When the data is broken down by income (as of June 2021), it shows that high-income countries (HICs) have already crossed the COVID-19 Vaccine Global Access (COVAX) 20% immunization threshold. However, countries of all other income levels have yet to achieve this mark for fully vaccinated people. Upper-middle-income countries (UMICs) have approximately 3%, low- and middle-income countries (LMICs) have approximately 2% and low-income countries (LICs) have less than 0.1% of fully vaccinated people per hundred. The supply shortage is expected to last until the second half of 2021.

**Conclusion:** As a result of the COVAX failure, a health gap emerged with countries living in a pre-immunization period for an extended time. The existing conflict between the international response to tackle COVID-19 and corporate profit-driven behavior contributed to prolonging pandemic, especially in Africa. Accordingly, there is a need to approve an international treaty that targets the activities of all actors, including the pharmaceutical companies, in protecting human rights and the right to health realms.

## Background

**Key Messages**

** Implications for policy makers**

This study prompts policy-makers to consider the following factors when developing policies nationally, regionally, and globally to secure equitable access to life-saving technologies:

- Nationally, countries, especially low- and middle-income countries (LMICs), should strengthen their productive capacities in the pharmaceutical sector to reduce dependence on foreign suppliers.
- Regionally, governments in LMICs can strengthen their cooperation mechanisms to improve epidemiological control and establish shared procurement instruments for essential medicines, vaccines, and other high-demand technologies.
- Globally, all governments should jointly demand more effective cooperation instruments and initiatives to reduce the risks of shortages of vital supplies during public health crises.
- Globally, all governments should demand an international pandemic response (binding) treaty that defines enforcement mechanisms that encompass responsibilities for governments, society, and corporations – clearly referring to pharmaceutical companies.

** Implications for the public**

This study underscores the challenges international organizations face when tackling severe public-health pandemics, specifically when regards to multilateral initiatives that rely on comprehensive multistakeholder collaboration. The most critical lesson is that society must demand the full realization of the right to health and hold not only national governments accountable but also the international community, organizations, and corporations, most notably pharmaceutical companies, to fulfill their particular responsibilities. Furthermore, it is crucial to understand that in a pandemic, such as coronavirus disease 2019 (COVID-19), global health security is only achieved by ensuring global and unrestricted access to all necessary technologies for everyone in all parts of the world. Thus, while tackling a pandemic, the main goal should be to leave no one behind.

The coronavirus disease 2019 (COVID-19) pandemic has killed more than six million people globally,^1^ leading to a severe social, economic, and humanitarian crisis with immeasurable effects – including increases in extreme poverty^2^ and hunger, resulting in starvation,^3^ sicknesses, and death; forced migration and displacements^4^; and worsened health conditions of marginalized and disproportionally affected populations. Thus, states, international organizations, civil society, and companies are tasked with addressing the pandemic and, in the process, establishing international obligations to strengthen human rights and, in particular, securing the right to health.

 Since the beginning of the coronavirus pandemic, the international community has responded to the outbreak of COVID-19 by building a comprehensive multilateral framework while reiterating the importance of mitigating the social and human costs that resulted from the severe acute respiratory syndrome coronavirus 2 (SARS-CoV-2) spread.^5^ The most ambitious and vital goal, involving national governments, international organizations, and pharmaceutical companies, was encouraging the production of effective and safe therapeutics and vaccines. To that end, several collaborative mechanisms such as the COVID-19 Technology Access Pool (C-TAP) and the COVID-19 Vaccine Global Access (COVAX) were launched in the first half of 2020 by multiple actors with different expertise, alongside making considerable resources available for subsidizing vaccine research and development (R&D) to accelerate the market registration of COVID-19 immunizations.

 Another critical aspect of building a global immunization system was ensuring that the most disproportionally affected populations had timely, adequate, and affordable access to innovative technologies developed to fight COVID-19, especially vaccines. In this regard, some essential multilateral resolutions were approved, and recommendations by international organizations were endorsed to ensure that COVID-19 immunization was conceived as a“global public good.” In May 2020, the World Health Organization (WHO) approved the “COVID-Response” Resolution (WHA 73.1), which proposed a global response to the pandemic, asserting the need for, “universal, timely, and equitable access to and fair distribution of health technologies and products to combat the virus.”^6^ To be effective, any unjustified obstacles to vaccine availability were to be removed. The United Nations General Assembly (UNGA) also approved two resolutions,^7,8^ emphasizing the need to scale manufacturing and strengthen supply chains rapidly. These efforts aimed to ensure efficient, timely, fair, transparent, and equitable access to and distribution of diagnostics, drugs, and COVID-19 vaccines to all of those in need, particularly in developing and least developed countries. As stated by United Nations (UN) Human Rights Bodies, equitable immunization was also recognized as a required effort to comply with international human rights obligations.^9,10^ Accordingly, the aim was to mitigate the harmful consequences of the coronavirus’s global spread without discrimination but prioritizing those who were most exposed to the risk of COVID-19.

 To accelerate the development of a vaccine and guarantee its fair distribution, some countries and philanthropic organizations started to fund the development of technologies associated with COVID-19 treatment and immunization. As two of the largest investors in COVID-19 vaccine R&D, the United States, and Germany contributed US $2 billion and US $1.5 billion, respectively. R&D investments totaled nearly US $5.9 billion until March 2021, with the vast bulk of it (approximately 98%) representing public funding. The Coalition for Epidemic Preparedness Innovations (CEPI), the United Kingdom, and the United States invested US $1. 7 billion to help create the AstraZeneca-Oxford vaccine and the US Government invested US $1.5 billion for the Johnson & Johnson vaccine. The CEPI, Dolly Parton, the COVID-19 Research Fund, and the United States invested US $957 million for the Moderna vaccine, and Germany invested US $957 million for the Pfizer-BioNTech vaccine.^11^

 Globally, the WHO has led efforts to develop and equally distribute vaccines through two unique mechanisms, the C-TAP^12^ and the COVAX.^13^ In May 2020, in collaboration with 42 Member States and implementing partners, the WHO launched C-TAP to voluntarily share knowledge and license intellectual property rights associated with COVID-19 responses. C-TAP aimed at increasing vaccine technology transfer and vaccine production, requesting engagement from governments and the pharmaceutical industry. This collaborative approach aimed to help accelerate COVID-19 vaccine developments and improve future readiness and production capacity. However, critics have pointed to the countries’ lack of commitment and the corporations’ outright reluctance to participate in C-TAP. Until the end of 2021, none of the countries with the technological developments in the area, including the US, the European Union, and India, have signed on and none of the vaccine-producing pharmaceutical corporations have joined C-TAP.^14^ Only in March 2022, the National Institutes of Health in the US announced that it would share the technology behind a coronavirus antibody test with C-TAP.^15^

 In April 2020, the WHO launched COVAX, a global cross-organization collaborative initiative for ensuring equitable vaccination distribution through a multilateral response mechanism financing affordable COVID-19 vaccines globally. Focusing on ensuring COVID-19 vaccines as a global public good, COVAX targeted helping low- and middle-income countries (LMICs), which account for roughly 85% of the world population and lack resources to purchase sufficient vaccine doses. To accomplish this objective, the COVAX Advanced Market Commitment, a specific finance scheme designed to ensure the availability of dosages for the poorest countries, was established. COVAX’s underlying premise was that, following global equality standards, no country should vaccinate more than 20% of its population until all nations have vaccinated 20% of their own populations.

 While COVAX appears to have achieved more success than the C-TAP in terms of being embraced by nearly every country in the world, critics have argued that wealthy countries have prioritized vaccinating their citizens at the expense of global vaccinations and that the pharmaceutical industry has focused on making the vaccine but not properly distributing it equally. The initiative’s goal of delivering 2 billion vaccine doses by the end of 2021 was unrealistic given the unequal distribution of vaccines, which have been drained by the high-income countries (HICs).

 These initial failures have placed severe concerns on the potential success of global immunization to combat COVID-19 and its global health consequences. Aiming to respond to the COVAX failure, the Indian and South African governments presented a waiver proposal for several World Trade Organization (WTO) Agreement on Trade-Related Aspects of Intellectual Property (TRIPS) obligations for all COVID-19 response technologies during the TRIPS Council meeting in October 2020 and then a revised version co-sponsored by other 65 countries.^16^ The core argument behind the waiver is that the enforcement of intellectual property rights is a critical obstacle to scale-up production to guarantee fair and equitable access for those disproportionally affected when voluntary and market-based initiatives fail. In June 2022, WTO members reached an agreement on the proposal. However, the decision is quite different from the original proposal, and is considered an ineffective compromise to address the issues raised by the waiver proponents.^17^

 Many recent studies have been dedicated to presenting the precarious state of global immunization, particularly the atrocious inequality in vaccine access.^18–20^ In this article, we analyze one of the structural causes of this problem - the corporate profit-driven discretion in vaccine dose allocation, drawing on the existing conflict between corporations and human rights and, more specifically, the responsibilities of companies not to violate human rights. One of the visible consequences of corporate autonomy and the tensions between profitability and the right to health was the COVAX failure. The debate^21^ on corporations and human rights is grounded in the adopted “Protect, Respect and Remedy: A Framework for Business and Human Rights” (UN Framework)^22^ and its “Guiding Principles on Business and Human Rights” (Guiding Principles).^23^ These documents clarified that companies are responsible for respecting human rights, which they define as a “do no harm” obligation independent of state duties. Specifically, regarding the pharmaceutical industry, “The Human Rights Guidelines for Pharmaceutical Companies in Relation to Access to Medicines” (Human Rights Guidelines for Pharmaceutical Companies)^24^ establishes that companies should refrain from taking unnecessary acts that jeopardize access to medicines. However, these documents and the literature^25^ on the pharmaceutical industry’s responsibility, which primarily focuses on the potential contradictions between intellectual property rights and access to medicines,^26^ do not address other profit-oriented behavior, including behaviors that directly affect the public interest and the right to health,^27^ such as corporate discretion to allocate life-saving immunizations.^28-30^

 The behavior of pharmaceutical companies raises questions about their responsibility for human rights violations in ensuring access to vaccines. The rhetoric of “solidarity” and “global public goods” seems to have been overcome by the harsh reality of *vaccine nationalism*^14,31^ in which governments prioritize their citizens at the expense of global health interests. This includes prohibiting vaccine and pharmaceutical supplies exports and the procurement of massive volumes of vaccine doses in advance through Advanced Purchase Agreements (APAs). APAs are legally binding contracts where governments agree to purchase a percentage of doses of a potential vaccine from a vaccine manufacturer at a negotiated price if the vaccine is produced, approved, and manufactured.

 Signing APAs appears to be the pharmaceutical companies’ top priority as they secure access to the most profitable markets, even though this commercial practice contradicts multilateral initiatives such as COVAX to tackle COVID-19. Therefore, the pharmaceutical industry’s maximalist behavior(pharmaceutical companies’ practices aimed at maximizing profits despite political commitments to protect human rights)has enabled vaccine nationalism and could be considered unresponsive to the need to guarantee universal access to vaccination.

 To analyze how corporate practices violate international human rights standards, the following research question was developed: to what extent has pharmaceutical companies’ behavior, primarily the signing of APAs, contributed to non-compliance with COVAX’s underlying premise of securing an equal 20% immunization threshold globally? To address this research question, this paper draws on the literature of commercial determinants of health,^32-34^ which in part examines corporate activities that are harmful to public health. We attempt to understand the impact of these commercial strategies on global health and the extent to which pharmaceutical companies’ maximalist conduct regarding vaccine dose distribution violates international multilateral commitments (eg, immunization against COVID-19), representing a fragility of the legal determinants of health.^35^ In doing so, we assess how their behavior infringed on corporate human rights legal standards by hampering access to a life-saving immunization.

## Methods

 We conducted a descriptive analysis of relevant data on multiple aspects of global vaccination to assess the implications of profit-driven decisions of pharmaceutical companies on the COVID-19 immunization program, particularly regarding the vulnerability of the COVAX initiative. Between December 2020 and June 2021, we reviewed COVID-19 vaccine data and the legal provisions that define the standards for corporate human rights responsibilities, and companies’ compliance with the right to health through access to medicines to assess their liability for possible violations of these rights. In particular, we reviewed the human rights legal framework’s documents, namely UN Framework, its Guiding Principles, and the Human Rights Guidelines for Pharmaceutical Companies.

 To understand vaccine allocation and multilateral commitments and initiatives’ objectives, we gathered and reviewed data from the “Our World in Data” database,^36^ a leading organization publishing global data on vaccinations for COVID-19 using interactive charts and maps. The organization is a project of the Global Change Data Lab, produced as a collaborative effort by researchers at the University of Oxford. These data are reasonably complete, accurate, and constantly updated. We analyzed data on the pattern of dose distribution according to regions and income, the volume of vaccine applications across countries, and the level of vaccination and of the immunized population. We then analyzed the countries that have yet to reach 5% of their population that have received at least one vaccination dose (Tables 1-3).

 We retrieved the schedule for dosage allocation from the COVAX official database through the information available on the GAVI Portal (COVAX Global Supply Forecast - 2021).^13^ We collected data on the doses delivered on a case-by-case basis using the GAVI Alliance Portal’s information in a specific (live) section. These data sought to assess if the COVAX schedule is being met or if the accumulated delays are significant enough to jeopardize the initiative’s strategic goals (Table 4).

 To better appraise distribution flows, we gathered data on the pharmaceutical companies’ commitment to COVAX, including the proportion of dosages made available to the initiative. We compiled data on the distribution of vaccine supplies produced and predicted to be produced in 2021, accounting for the volume going to countries based on their income. We also reviewed WHO and CEPI data and the manufacturers’ press releases and other communications on COVAX supply agreements to examine the factors that contributed to the COVAX shortage.^37^ We also collected and analyzed data on APAs between governments (mostly HICs) and pharmaceutical companies. We reviewed data on APAs from the Duke Global Health Innovation Center (The Launch and Scale Speedometer^38^) – a tool aimed at thoroughly evaluating the variables that support or hinder the introduction and scaling up of interventions to solve critical global health issues – to determine to which extent the agreements impacted corporate accountability to COVAX and, as a consequence, the approved multilateral resolutions that demand a worldwide commitment to timely and equitable immunization (Tables 5 and 6).

 The data compiled and analyzed served as the foundation for evaluating the health risks associated with the behavior adopted by pharmaceutical companies during the ongoing pandemic. In addition, we learned about the limitations and legal implications of multilateral initiatives to tackle COVID-19 by examining the instruments that conform to the international legal apparatus. Moreover, we built the basis to analyze corporate liability for human rights and compliance with the right to health.

 To cover the period between December 2020 and June 2021, we did the final data gathering in the first week of July 2021 from the various databases. Therefore, we built the tables based on this period. However, in the first week of June 2022, we updated part of the collected data. Accordingly, we updated the information on the COVAX goal of delivering 2 billion doses by the end of 2021 in the background and discussion sections. In addition, the background and discussion sections have also updated information regarding COVAX delivery numbers and vaccine prices. Similarly, we have updated the extreme inequality scenarios, comparing HICs to low-income countries (LICs). We emphasized the discrepancy between LICs and HICs, including data primarily from the United States, Europe, and Canada, on the one hand, and the African continent, on the other hand. Since all of the data was publicly available, no ethical consent was required.

## Results

###  Lack of Vaccine Distribution

 By the end of 2021, ten major pharmaceutical companies were expected to deliver more than 12 billion vaccine doses.^38^ If distributed equitably,^33^ this volume of vaccine doses would have been sufficient to approach a global herd immunity.However, these expectations fell short of achieving a balanced distribution. Tables 1 and 2 show the total number of people who have received at least one dose of the COVID-19 vaccine, and the volume of the population who has been fully vaccinated (who have received all of the doses prescribed by the specific vaccination protocol) per 100 people by region and income level.

 The figures in Table 1 demonstrate the significant disparity in the volume of vaccines administered in different world regions (as of June 2021). Not even two people have been vaccinated for every 100 people on the African continent, although other regions (eg, European Union and North America) have achieved significant progress in vaccination rates (approximately 39.3% and 52.1% per hundred people, respectively). The European continent, including Eastern European countries where vaccination is still progressing at a slower pace due to supply problems in the European Union, has already reached more than 32% of people who have received at least one dose of an authorized vaccine. Asia and South America lag behind Europe and are far behind the European Union and North America. Although there are HICs on the Asian continent, the aggregated data show how the Global South has been vulnerable when it comes to vaccine access. These data reveal that a massive percentage of the world’s population lives in a health situation of absolute exclusion, posing severe risks to global health, whereas other regions are transitioning to a post-pandemic phase.

**Table 1:** Vaccinated and Immunized Population by Region

**Region**	**Last Update**	**People Vaccinated Per Hundred**	**People Fully Vaccinated Per Hundred**
Africa	06/02/2021	1.87	00.66
Asia^a^	06/02/2021	6.16	02.31
South America	06/02/2021	19.25	09.34
Europe	06/02/2021	32.36	17.47
European Union^b^	06/02/2021	39.32	19.28
North and Central America	06/02/2021	37.62	26.19
North America (Canada, USA)	06/02/2021	52.17	38.01

^a^Asia includes all countries part of the continent, as Southeast Asia figures are not detailed separately. ^b^Statistics from the European Union are shown to emphasize the higher rates in western European countries.

 As indicated in Table 2, the lack of access to vaccines is observed in LICs. When the data is broken down by income (as of June 2021), it shows that HICs have already crossed the 20% mark for fully vaccinated people. However, countries of all other income levels have yet to achieve the COVAX threshold of 20% (upper-middle-income countries [UMICs] approximately 3%; LMICs approximately 2% and LICs less than 0.1% of fully vaccinated people per hundred), and with significant supply shortages expected to last until the second half of 2021. Accordingly, the dosage distribution flow does not fulfill international equity criteria established in international resolutions (WHA 73.1, UNGA 74/270, and UNGA 74/274).

**Table 2:** Vaccinated and Immunized Population by Income

**Income**	**Last Update**	**People Vaccinated Per Hundred**	**People Fully Vaccinated Per Hundred**
HIC	06/02/2021	36.50	22.47
UMICs	06/02/2021	06.30	03.95
LMICs	06/02/2021	07.22	02.20
LICs	06/02/2021	00.70	<00.10

Abbreviations: HICs, High-income countries; UMICs, upper-middle-income countries; LMICs, low- and middle-income countries; LICs, low-income countries.

 More recent data (June 2022) show that this pattern of disparity has not changed. LICs have not yet reached 15% of the population fully vaccinated. In contrast, HICs and UMICs have not completed full vaccination due to the hesitancy of the population to be vaccinated.^39^ Likewise, the African continent continues to experience the most significant vaccine shortages. While the world has reached 66% of people vaccinated with at least one dose, this number is only 23% for the African continent.

Table 3 further examines the situation in a portion of the world’s population that is still underserved. As illustrated, 68 countries have yet to reach 5% of their population that has received at least one vaccination dose. Only two of these nations (Thailand and Belarus) are not participants of the COVAX initiative, while 13 others have joined as self-financing members. In other words, 53 of the 68 countries with the poorest immunization rates are AMC-eligible (Advanced Market Commitment for COVID-19 vaccine access) and rely on COVAX to guarantee vaccine supplies. More than half of these countries (38 countries) are supported by the WHO African Office, while the WHO Regional Office supports another 10 for the Eastern Mediterranean, which comprises North African and Middle Eastern countries. Linked to the Pan American Health Organization (PAHO), Latin American countries perform relatively better, with only five countries on the list - and only two of them are AMC-eligible.

**Table 3:** Unassisted Populations

**Countries**	**People Vaccinated Per Hundred**	**WHO Regional Office**	**Income**	**COVAX Status**
Central African Republic	<0.1	AFRO	LIC	AMC
Democratic Republic of Congo	<0.1	AFRO	LIC	AMC
South Sudan	<0.1	AFRO	LIC	AMC
Yemen	<0.1	EMRO	LIC	AMC
Madagascar	<0.1	AFRO	LIC	AMC^a^
Benin	0.1	AFRO	LMIC	AMC
Papua New Guinea	0.1	WPRO	LMIC	AMC
Cameroon	0.2	AFRO	LMIC	AMC
Guinea-Bissau	0.3	AFRO	LIC	AMC
Congo	0.4	AFRO	LIC	AMC
Mali	0.4	AFRO	LIC	AMC
Mauritania	0.5	AFRO	LMIC	AMC
Sudan	0.5	AFRO	LIC	AMC^a^
Gabon	0.5	AFRO	UMIC	SF^a^
Niger	0.6	AFRO	LIC	AMC
Kyrgyzstan	0.6	EURO	LMIC	AMC
Sierra Leone	0.7	AFRO	LIC	AMC
Armenia	0.7	EURO	UMIC	SF
Somalia	0.8	AFRO	LIC	AMC
Zambia	0.8	AFRO	LMIC	AMC
Tajikistan	0.8	EURO	LIC	AMC
Nigeria	0.9	AFRO	LMIC	AMC
Mozambique	1.0	AFRO	LIC	AMC
Vietnam	1.0	WPRO	LMIC	AMC
Gambia	1.1	AFRO	LIC	AMC
Liberia	1.1	AFRO	LIC	AMC
South Africa	1.1	AFRO	UMIC	SF
Iraq	1.1	EMRO	UMIC	SF
Venezuela	1.1	PAHO	UMIC	SF
Uganda	1.2	AFRO	LIC	AMC
Afghanistan	1.2	EMRO	LIC	AMC
Djibouti	1.3	EMRO	LMIC	AMC
Ethiopia	1.4	AFRO	LIC	AMC
Honduras	1.4	PAHO	LMIC	AMC
Guinea	1.6	AFRO	LIC	AMC
Libya	1.6	EMRO	UMIC	SF
Lesotho	1.7	AFRO	LMIC	AMC
Kenya	1.8	AFRO	LMIC	AMC
Malawi	1.8	AFRO	LIC	AMC
Egypt	1.8	EMRO	LMIC	AMC
Cote d'Ivoire	1.9	AFRO	LMIC	AMC
Pakistan	1.9	EMRO	LMIC	AMC
Angola	2.0	AFRO	LMIC	AMC
Guatemala	2.1	PAHO	UMIC	SF
Ukraine	2.3	EURO	LMIC	AMC
Nicaragua	2.5	PAHO	LMIC	AMC
Namibia	2.5	AFRO	UMIC	SF^b^
Senegal	2.6	AFRO	LMIC	AMC
Ghana	2.7	AFRO	LMIC	AMC
Rwanda	2.7	AFRO	LIC	AMC
Kosovo	2.9	EURO	UMIC	AMC^c^
Eswatini	3.0	AFRO	LMIC	AMC
Georgia	3.0	EURO	UMIC	SF
Botswana	3.0	AFRO	UMIC	SF^b^
Thailand	3.2	SEARO	UMIC	-
Philippines	3.2	WPRO	LMIC	AMC
Togo	3.3	AFRO	LIC	AMC
Myanmar	3.3	SEARO	LMIC	AMC
Iran	3.3	EMRO	UMIC	SF
Bangladesh	3.5	SEARO	LMIC	AMC
Paraguay	3.5	PAHO	UMIC	SF
Uzbekistan	3.6	EURO	LMIC	AMC
Cape Verde	3.9	AFRO	LMIC	AMC
Oman	4.2	EMRO	HIC	SF
Zimbabwe	4.3	AFRO	LMIC	AMC
Belarus	4.6	EURO	UMIC	-
Tunisia	4.7	EMRO	LMIC	AMC
Timor	4.8	SEARO	LMIC	AMC

Abbreviations: WHO, World Health Organization; COVAX, COVID-19 Vaccine Global Access; HIC, high-income country; UMIC, upper-middle-income country; LMIC, low- and middle-income country; LIC, low-income country; AFRO, African region; EMRO, Eastern Mediterranean region; EURO, European region; PAHO, Pan American Health Organization; SEARO, South-east Asian region; WPRO, Western Pacific region; SF, self-financing; AMC, Advanced Market Commitment for COVID-19 vaccine access. ^a^ Countries eligible as AMC or self-financing but which have not yet had doses allocated by COVAX. ^b^ Some of the potentially self-financing countries that have expressed written interest in the COVAX initiative have requested that their names be kept confidential. Thus, we assumed that countries receiving doses but not listed as AMC eligible are self-financing. ^c^ World Bank International Development Association (IDA)-eligible economies.

 When the data is broken down by country, it becomes evident that the African continent is still operating in the pre-vaccine era, as if there is no immunization mechanism in place to prevent the coronavirus from spreading. When we contrast data from African countries with data from regions and countries with upper ranks of immunization, the continent’s serious sanitarian situation and level of inequality become even more striking. Several countries have yet to launch vaccination campaigns, and several others have yet to reach the 1% mark of people who have received at least one dose of the vaccine.

###  Vaccine Allocation, Delivery, and COVAX Delays

 According to GAVI Alliance COVAX Forecast, in the first quarter of 2021, the first and second rounds of allocation indicated a distribution of 237 million doses of the AstraZeneca-Oxford vaccine and 1.2 million doses of the Pfizer-BioNTech vaccine. These rounds of allocation intended to ensure that all countries, regardless of their development or economic condition, received a share of the available doses through COVAX. Each country part of COVAX was assigned doses based on public health objectives for COVID-19 control, taking into account epidemiological characteristics and vulnerabilities, among other factors. However, allocation does not always imply delivery of vaccination doses, and the pharmaceutical industry failed to meet the COVAX Forecast set by the GAVI Alliance. Accordingly, the doses supposedly allocated to each WHO regional office were never fully delivered due to lack of availability since the industry compromised the largest share of their production supplying HICs – complying with APAs.

Table 4 demonstrates the challenges faced by the COVAX initiative in distributing the vaccine doses expected to be delivered by May 2021 (the first and second rounds). We confirmed that the COVAX initiative did not meet the announced forecast for delivery when we integrated the figures from Pfizer-BioNTech and AstraZeneca-Oxford’s actual delivery of doses. A little more than 32 million doses were actually delivered out of a total of 167 million doses initially agreed to supply WHO regional offices, accounting for roughly 20% of the total. As detailed in Table 4, of the 167 million doses, the WHO-EURO and PAHO regional offices could only deliver 26% of the number of doses initially agreed for each office in the first and second rounds. WHO-AFRO regional office delivered 22%, WHO-EMRO 18%, and WHO-SEARO/WRPO 12%. Thus, the problem was not in their distribution (since the WHO regional offices distributed in a fair and equitable manner), but rather structural, since COVAX only received 20% of the total expected.

**Table 4:** COVAX Supply and Delays

**WHO Regional Office**	**Doses Allocated (First and Second Rounds)**	**Doses Delivered (by May 31)**	**Doses Delivered (%)**
WHO-AFRO	59 024	12 964	22%
WHO-PAHO	28 824	7436	26%
WHO-EMRO	23 203	4207	18%
WHO-EURO	7019	1839	26%
WHO-SEARO/WRPO	49 510	5787	12%
**Total**	167 580	32 233	20%

Abbreviations: COVAX, COVID-19 Vaccine Global Access; WHO, World Health Organization; AFRO, African region; EMRO, Eastern Mediterranean region; EURO, European region; PAHO, Pan American Health Organization; SEARO, South-east Asian region; WPRO, Western Pacific region.

###  Factors Contributing to COVAX Uneven Distribution

 Several HICs, including the United States, Canada, European Union countries, Israel and a few middle-income countries, including Brazil, Mexico, and Turkey, signed APAs to guarantee enough vaccine doses to immunize their citizens several times before the vaccines were approved for commercialization. Until 2021, when most of the COVID-19 vaccines were approved for commercialization, the mentioned countries had signed at least 45 APAs, led by the United States and the United Kingdom with nine agreements each, Canada with a total of eight agreements, the European Union with seven, and Israel with five agreements. In addition, Brazil and Mexico signed two agreements each while Turkey signed three. This allowed these countries priority access to available vaccines and the ability to achieve much higher immunization rates (Israel 60%, UK 49%, US 46%, Canada 31%, European Union 33.6%, Turkey 18%, Mexico 15%, Brazil 12%) by June 2021.^38^ This also allowed these countries to continue to procure extra vaccine dosages in 2021.

 The COVAX portfolio consists of six vaccines: The Oxford/AstraZeneca, Pfizer-BioNTech, Moderna, Janssen, Novavax, and, as of May 2021, the Sinopharm vaccine. The Sputnik V, produced by the Gamaleya Institute, has not been approved by the WHO, while the Coronavac vaccine, produced by Sinovac, was approved by the WHO in June 2021, but there is no information on their commitment to supplying the COVAX consortium as of July 2021. According to data from Duke University’s Global Health Innovation Center, AstraZeneca-Oxford (committed in June 2020), Janssen (committed in December 2020), and Pfizer-BioNTech (committed in January 2021) made early-to-mid commitments to COVAX while Moderna (committed in May 2021) made a mid-to-late commitment. However, as stated in Table 5, they only contributed a symbolic-to-small percentage of their vaccine production compared to the volume of doses distributed to a few HICs. Moderna, AstraZeneca-Oxford, and Janssen agreed to contribute 37%, 27%, and 23% of their production respectively to supply COVAX, while Pfizer only contributed 1.5% while offering 79% of their doses to HICs. Countries in this group are estimated to have purchased approximately six billion doses.^33^ In contrast, LICs and LMICs have guaranteed fewer doses through anticipated purchases, and the COVAX participants – including all 92 low-and-middle-income countries – will only have access to approximately 2.4 billion doses.^35^

**Table 5:** Vaccine Procurement and Supply Agreement With COVAX

**Income - COVAX**	**AstraZeneca-Oxford**	**Janssen**	**Pfizer-BioNTtech**	**Moderna**
HICs	965 400 (37%)	352 000 (41%)	239 6870 (79.0%)	816 500 (62.0%)
UMICs	498 140 (19%)	59 100 (8%)	510 100 (17.0%)	1050 (>0.1%)
LMICs + LICs	429 725 (17%)	240 000 (28%)	76 400 (2.5%)	13 000 (>1.0%)
COVAX	721 000 (27%)	200 000 (23%)	40 000 (1.5%)	500 000 (37.0%)
**Total**	2 614 265 (100%)	851 100 (100%)	3 023 370 (100%)	1 330 550 (100%)

Abbreviations: COVAX, COVID-19 Vaccine Global Access; HICs, High-income countries; UMICs, upper-middle-income countries; LMICs, low- and middle-income countries; LICs, low-income countries.

 HICs injected public funds into COVID-19 vaccine R&D and used their purchasing power to negotiate large-scale deals on various vaccine candidates. As Table 6 depicts, 10 of the 16 top vaccine buyers are HICs (Canada, UK, New Zeeland, Australia, Chile, Israel, USA, Switzerland, South Korea, and Japan) along with the European Union. Except for Bolivia and Morocco, these top buyers are not AMC eligible (they are either donors or self-financing) and signed most of the APAs with pharmaceutical companies that expressed an early-to-mid commitment to COVAX (AstraZeneca-Oxford, Janssen, and Pfizer-BioNTech). As these companies prioritized supplying the top buyers, allocating vaccine doses up to approximately 10 times their population they were not able to comply with what they had initially agreed to allocate to the COVAX initiative. For example, Canada, the United Kingdom, and Australia purchased approximately eight, seven and six doses per inhabitant, respectively. Export restrictions enforced by several countries to guarantee that vaccine doses were delivered first to domestic needs have exacerbated the problem.

**Table 6:** Vaccine Procurement

**Countries**	**Income Level**	**Dose Purchase Per Inhabitant**	**Total Doses Purchase** **(Million)**	**Pfizer** **(Million)**	**Oxford-Astraz.** **(Million)**	**Moderna** **(Million)**	**Janssen** **(Million)**	**Novavax** **(Million)**	**Sanofi-GSK** **(Million)**	**Others (Gamaleya, Sinopharm, Sinovac) (Million)**	**COVAX Status**	**COVAX - Doses Allocated (Million)**
Canada	HIC	10.44	364	105	23	44	10	52	72	0	Self-financing	1.7
UK	HIC	8.18	517	100	100	17	30	60	60	0	Self-financing	0.5
EU	HIC	6.89	2850	1500	300	310	200	0	300	0	Donor	0
New Zeeland	HIC	6.57	30	10	8	0	2	11	0	0	Self-financing	0.3
Australia	HIC	5.82	170	40	53	25	0	51	0	0	Self-financing	0.5
Chile	HIC	5.07	90	10	14	0	0	0	0	60.0	Self-financing	0.8
Israel	HIC	4.53	41	17	10	10	0	0	0	0	Self-financing	0
USA	HIC	3.99	1200	300	300	300	100	110	100	0	Donor	-
Switzerland	HIC	3.67	31	6	0	20	0	0	0	0	Self-financing	0
South Korea	HIC	3.44	172	66	20	40	6	40	0	0	Self-financing	0
Bolivia	LMIC	3.40	24	0	5	0	15	0	0	4.5	AMC	0.2
Japan	HIC	2.88	364	194	120	50	0	0	0	0	Self-financing	0
Turkey	UMIC	2.57	214	64	0	0	0	0	0	50.0	Not involved	-
Morocco	LMIC	2.50	91	0	50	0	0	0	0	0	AMC	1.7
Brazil	UMIC	2.31	450	200	102	0	38	0	0	10.0	Self-financing	5.0
Malaysia	UMIC	2.18	66	25	6	0	0	0	0	6.0	Self-financing	1.4

Abbreviations: COVAX, COVID-19 Vaccine Global Access; HIC, High-income country; UMIC, upper-middle-income country; LMIC, low- and middle-income country; LIC, low-income country; AMC, Advanced Market Commitment for COVID-19 vaccine access.

## Discussion

 The multilateral provision for extensive global access to COVID-19 vaccinations, which is crucial for controlling the spread of the virus and reducing the mortality rate, has been threatened by pharmaceutical companies’ maximalist approach. Despite initiatives to address vaccine access, such as the launch of COVAX, non-compliance with the initiative’s threshold is not helping to address the equitable approach to establishing global herd immunity but instead is widening the access gap, especially in poorer regions of the world.

 The COVAX initiative seems so jeopardized that WHO Director Tedros Adhanom Ghebreyesus stated that the COVAX equitable distribution system is at “serious risk” due to the profound imbalance in the global distribution of vaccines.^40^ Amid a pandemic with profound health implications, some pharmaceutical companies have been hesitant to commit to COVAX, while others have committed to providing only a fraction of vaccine doses relative to their capacity. Likewise, other companies that have agreed to allocate doses to assist the WHO’s distribution strategy have prioritized selling it to countries that offered higher prices, only committing to delivering the vaccine doses in the initiative’s mid-late or late stage. Consequently, the COVAX initiative suffers from shortages and has missed its first deadline (May 31, 2021)^41^ for delivering doses to eligible countries, according to GAVI official forecast. One year later, in May 2022, the initiative has not reached the goal of delivering 2 billion doses yet, as the initiative just reached 1.5 billion doses delivered to 145 countries and territories. Accordingly, due to insufficient dose delivery, many countries and hundreds of millions of people will only have access to vaccines in late 2024.^42^

 This experience raises concerns about fulfilling the right to health through equitable access to life-saving immunization, shifting the attention to pharmaceutical companies’ responsibilities towards human rights standards and compliance with the multilateral legal framework to fight COVID-19. Accordingly, the lack of vaccines in the world sounded the alarm about corporate liability to potential health risks, calling for the advancement of legal mechanisms to enforce companies’ accountability for human rights regulations while conducting public functions such as the allocation of vaccines during a global health crisis.

 The commercial and legal determinants of health are at the center of this debate. This study has helped contribute to this literature as the analyzed data underlines the link between companies’ profit-driven conduct and COVAX delays, shedding light on its effects on the pandemic time frame and population health. Furthermore, issues raised by pharmaceutical companies’ maximalist behavior are seldom treated as an integral part of analyses using commercial determinants of health frameworks. This is especially relevant given that companies’ profit-driven approach directly affects the dynamics of several determinants of health, such as access to life-saving immunization. In the same line, the results add to the debate on legal determinants of health, attesting to the vulnerability of international recommendations and formal instruments – especially the lack of authoritative and enforcement mechanisms of international norms that establish the legal framework for the right to health and guarantee of human rights.

 In this sense, and considering the seriousness of the problem, it is necessary to better integrate the different frameworks to secure the fulfillment of human rights. Advancement of legally binding treaties that address corporate activities and compliance with multilateral initiatives to address global health problems could be a feasible solution to minimize the impact of pharmaceutical companies’ maximalist approach on securing equitable access to life-saving medicines. Furthermore, the present pandemic has highlighted the importance of legal reform to remove commercial hurdles to adopting multilaterally developed schemes aimed at assuring access to the most disproportionally affected populations.

 The UN Framework and its Guiding Principles are intended to serve as an authoritative focal point and reference for companies to rely on and align their actions to increase compliance with their human rights obligations. Based on these documents, there is room to argue that the profit-maximizing conduct utilized by vaccine manufactures during the COVID-19 vaccine race resulted in an unquestionable concentration of vaccine doses in HICs. Thus, the world faces a lack of vaccines, with completely unassisted regions. Moreover, since the data on the shortage of vaccines illustrated imminent health and human harm to a significant portion of the population, with a rising global health risk as a result of the lengthening of the pandemic and the emergence of new virus variants. This analysis points to an absence of positive steps by the pharmaceutical companies to avoid human rights violations and mitigate damage by properly supplying COVAX. It is worth mentioning that most variants have emerged in LMICs, where vaccine access has proven to be limited.^43^

 The UN Framework and its Guiding Principles, and the decision of the pharmaceutical companies to prioritize the selling of their vaccines to a selective group of countries through private procurement strategies, indicate that they have jeopardized LMIC’s attempts to protect human rights. This statement is embedded in the fact that these two documents establish that (*a*) companies must be guided by their social function and not just their material interests. This is fundamental to frame the performance and responsibilities of companies in critical situations. In the case under analysis, allocating life-saving immunization during a global pandemic is the social function of pharmaceutical companies; (*b*) the analyzed pharmaceutical companies have voluntarily committed to supplying COVAX, creating legitimate expectations for countries relying on it, as explained in the UN Framework and its Guiding Principles and; (*c*) the COVAX initiative was the main instrument created by the international community, based on general principles of cooperation and solidarity and specific rules for the promotion of global health and the guarantee of human rights. Therefore, the intentional distortion of COVAX functions and the abandonment of the voluntary commitments could be seen as violations of the UN Framework and its Guiding Principles and the approved multilateral resolutions.

 In the same line, but dealing specifically with the social role of pharmaceutical companies, society also has legitimate expectations of the companies’ behavior when holding the patent on a life-saving vaccine. The international agreements that define the rules for the protection of intellectual property rights (more precisely, article 8 of the TRIPS Agreement) and the Doha Declaration on the TRIPS and Public Health establish that intellectual property rights must not be an obstacle to guaranteeing access to health.^44–46^ So, private rights over a life-saving vaccine and the right to health must be balanced. This is precisely what was determined at the multilateral level to fight COVID-19. In this sense, the UN Framework and its Guiding Principles and the resolutions adopted to fight the coronavirus placed decisive right-to-health responsibilities on companies, which must act in a socially responsible way. Thus, the Human Rights Guidelines for Pharmaceutical Companies support a case to be made that the profit-driven approach of pharmaceutical companies has also contradicted the mentioned document. This argument is grounded in the fact that the listed companies did not act to guarantee that the vaccines were allocated and delivered – securing affordability – to as many countries as possible, hampering COVAX’s objective to ensure a fair and equitable distribution of COVID-19 immunization. This argument becomes even more robust as data illustrates that a significant portion of public funds subsidized COVID-19 R&D.

 It can also be noted that COVAX does not challenge pharmaceutical companies because its Voluntary mechanism leaves room for companies to decide the extent of their commitment. Thus, the absence of any enforcement mechanism makes it impossible to ensure that COVAX deadlines are met. There are solid reasons to sustain the argument that the TRIPS waiver would encourage the entering of new suppliers into the COVID-19 vaccine market, resulting in lower prices and pressure to increase production and licensing of already developed vaccines.^47-49^ In this sense, the proposal shares the expectations of a fairer and equitable distribution of the COVID-19 vaccines, but not COVAX’s ideal of volunteerism. The proposal asks for a waiver of patents, copyright, industrial design, and protection of undisclosed information and the application of the TRIPS enforcement section to all technologies applied in the diagnosis, immunization, and treatment of COVID-19. More than two years after WHO declared COVID-19 as a global pandemic, WTO members reached a formal agreement. The decision is significantly less ambitious than the initial proposal presented by India and South Africa. It covers only vaccines, keeping aside therapeutics and diagnostic tests. Also, it applies exclusively to patent rights and suggests limiting the countries that could implement the flexibilities. Accordingly, the decision minimizes the potential for permanent technical cooperation between companies to share and transfer technologies.

 Through the exercise of intellectual property rights, pharmaceutical companies can control the entry of new producers into the market. Pharmaceutical companies can also limit who can produce vaccines, the volume of vaccine doses, the prices charged, and whom to sell to through their APAs. This power creates barriers to increasing of vaccine production. This power becomes even more critical given the substantial role governments play in HICs, developing countries, international organizations, and large philanthropic organizations in funding the development of technologies applied to the production of vaccines for COVID-19.

 Another problem that results from the discretionary power of pharmaceutical companies in allocating the doses produced and controlling the limits of technology transfer is precisely the negligence concerning COVAX. The fact that the agreements signed with the initiative provide fixed prices lower than those freely practiced in the market leads companies to direct their sales to HICs at first because they can pay higher prices and still purchase large volumes. To illustrate this problem, UNICEF Vaccine Market Dashboard has compiled some data on prices charged by some pharmaceutical companies. For example, Moderna set a price of $7 per dose for COVAX while charging $15 from the US government and between $18 and $35 from the European Union. On average, the price charged for HICs was $32. On the other hand, the price difference practiced by Janssen is not that wide, ranging from 7.5 for COVAX and 10 dollars for the United States.^50^

 The weakening of COVAX leaves LICs dependent on donations,^51^ or leads them to seek individual solutions, but the prices charged by pharmaceutical companies become another problem. AstraZeneca, Moderna, and Pfizer are charging different prices to different countries, and HICs have been paying lower prices while LICs pay twice the price.^52,53^ As a result, most African countries face the devastating consequences of limited access to COVID-19 vaccines, including the collapse of the healthcare system.^54^

 These findings raise the alarm about the pharmaceutical companies’ behavior and partially explain the crises LMICs face through the COVID-19 pandemic. Accordingly, pharmaceutical companies that own the technologies to produce the currently authorized vaccines have many concerns about the waiver, resulting in their constant lobby to prevent its approval.^55,56^

 The descriptive analysis presented in this study confirms the urgency to create an international treaty that specifically addresses and regulates the activities of pharmaceutical companies in the area of human rights. This is currently a central issue for the Human Rights Council Open-ended Intergovernmental Working Group, which aims to build upon the UN Framework and its Guiding Principles to draft a legally binding instrument to regulate the activities of transnational corporations’ activities and other businesses.^57^ During the Working Group’s sixth session – October 2020, the UN High Commissioner for Human Rights emphasized the centrality of the instrument to the lives and livelihoods of the world population, especially in critical times such as the current health crisis.^9^ Furthermore, companies’ right to free exercise of economic activities should be questioned and limited when the risk imposed on global health is imminent and infringes on the right to health.

 Human rights and public health advocates should draw on other international efforts to protect public health globally. This includes drawing on the experience of the Framework Convention on Tobacco Control (FCTC), the first and only international public health treaty, which has been signed and ratified by more than 150 countries.^58^ The FCTC helps regulate the political and marketing practices of the tobacco companies, and FCTC Article 5.3 specifically removes tobacco companies as stakeholders in the decision-making process, thereby diminishing their influence in protecting public health. This also includes drawing on the Framework Convention on Global Health, which is drafted as a global health treaty based on the right to health to establish national and global health equity.

 Finally, this study is not absent of limitations. As it is a descriptive analysis, no correlation can be drawn from the signing of APAs and COVAX delays. Thus, we can only indicate that it was one of the factors that jeopardized COVAX.

## Conclusion

 Due to the COVAX failure, the health gap is increasing daily, with several countries in the Global South and vast population continents living in a pandemic situation for an extended time. Furthermore, by prioritizing the sale of their vaccines to HICs through signing APAs, pharmaceutical companies’ behavior contributed to the inability of COVAX to reach its immunization threshold. As a result, COVAX has not been able to keep its schedule of vaccine deliveries to countries and populations that depend on this multilateral initiative to advance their immunization policies. With this, the world is experiencing an unequal vaccination dynamic. Nevertheless, there were sufficient material resources to enable fair and equal access to vaccines by the end of 2021, hampered only by commercial interests, which is a serious breach of fundamental human rights and creates severe risks to global health. Based on the results, we can infer that the multilateral response to tackle COVID-19 has impactful drawbacks, mainly as it lacks mechanisms to enforce compliance with proposed initiatives such as COVAX. Accordingly, we draw attention to the need for approving an international treaty – having the FCTC as a precedent – that targets the activities of all actors. This includes the pharmaceutical companies, in the human rights and the right to health realms, particularly concerning access to life-saving technologies in critical situations, such as the current pandemic.

## Acknowledgements

 We thank the University of Nevada, Reno, the Brazilian National Council for Scientific and Technological Development (MCTIC/CNPq Nº 28/201), and the Fulbright Commission (Fulbright-CAPES Scholarship, ME/CAPES Nº 8/2020). However, neither the university nor other institutions played a role in conducting the research or preparing this article.

## Ethical issues

 Since all of the data was publicly available, no ethical consent was required.

## Competing interests

 Authors declare that they have no competing interests.

## Authors’ contributions

 LCB and HZM conceptualized the study and collected the raw data. LCB prepared the first and subsequent drafts of the manuscript. HZM and EC contributed to revisions of the paper.
